# Real-World Effectiveness of Nirmatrelvir in Protecting Long COVID for Outpatient Adult Patients – A Large-Scale Observational Cohort Study from the RECOVER Initiative

**DOI:** 10.21203/rs.3.rs-4536807/v1

**Published:** 2024-06-20

**Authors:** Fei Wang, Chengxi Zang, Haoyang Li, Dhru Khullar, Yongkang Zhang, Stephenson Strobel, Yong Chen, Marc Sala, Payal Patel, Alejandro Comellas, Andrew Wylam, Mark Weiner, Christopher Forrest, Thomas Carton, Rainu Kaushal

**Affiliations:** Weill Cornell Medicine; Weill Cornell Medicine; Weill Cornell Medicine; Weill Cornell Medicine; Weill Cornell Medicine; Weill Cornell Medicine; Department of Biostatistics, Epidemiology and Informatics, University of Pennsylvania; Northwestern University; University of Washington School of Medicin; University of Iowa; 6Patient representative for Long COVID patients with the National Institutes of Health’s RECOVER Initiative; Weill Cornell Medicine; Children’s Hospital of Philadelphia; Louisiana Public Health Institute; Weill Cornell Medicine, New York, NY

## Abstract

Paxlovid has been approved for use in patients who are at high risk for severe acute COVID-19 illness. Evidence regarding whether Paxlovid protects against Post-Acute Sequelae of SARS-CoV-2 infection (PASC), or Long COVID, is mixed in high-risk patients and lacking in low-risk patients. With a target trial emulation framework, we evaluated the association of Paxlovid treatment within 5 days of SARS-CoV-2 infection with incident Long COVID and hospitalization or death from any cause in the post-acute period (30–180 days after infection) using electronic health records from the Patient-Centered Clinical Research Networks (PCORnet) RECOVER repository. The study population included 497,499 SARS-CoV-2 positive patients between March 1, 2022, to February 1, 2023, and among which 165,256 were treated with Paxlovid within 5 days since infection and 307,922 were not treated with Paxlovid or other COVID-19 treatments. Compared with the non-treated group, Paxlovid treatment was associated with reduced risk of Long COVID with a Hazard Ratio (HR) of 0.88 (95% CI, 0.87 to 0.89) and absolute risk reduction of 2.99 events per 100 persons (95% CI, 2.65 to 3.32). Paxlovid treatment was associated with reduced risk of all-cause death (HR, 0.53, 95% CI 0.46 to 0.60; risk reduction 0.23 events per 100 persons, 95% CI 0.19 to 0.28) and hospitalization (HR, 0.70, 95% CI 0.68 to 0.73; risk reduction 2.37 events per 100 persons, 95% CI 2.19 to 2.56) in the post-acute phase. For those without documented risk factors, the associations (HR, 1.03, 95% CI 0.95 to 1.11; risk increase 0.80 events per 100 persons, 95% CI −0.84 to 2.45) were inconclusive. Overall, high-risk, nonhospitalized adult patients with COVID-19 who were treated with Paxlovid within 5 days of SARS-CoV-2 infection had a lower risk of Long COVID and all-cause hospitalization or death in the post-acute period. However, Long COVID risk reduction with Paxlovid was not observed in low-risk patients.

## Introduction

Some patients who recover from the acute phase of COVID-19 infection develop new, prolonged, or exacerbated medical conditions, often referred to as Post-Acute Sequalae of SARS-CoV-2 Infection (PASC) or Long COVID.^1–3^ No standard definition or diagnostic criteria exists for Long COVID, and researchers are still clarifying the biological mechanism through which these conditions develop.^3^

Although no treatments for Long COVID have been approved by the U.S. Food and Drug Administration, there is growing interest in whether the antiviral, nirmatrelvir-ritonovir (Paxlovid), could mitigate the long-term consequences of SARS-CoV-2 infection. Paxlovid received emergency use authorization (EUA) in December 2021 for treatment of non-hospitalized COVID-19 patients with mild to moderate acute infection who are at high risk of progressing to severe COVID-19 (treatment recommended within 5 days of symptom onset)^4,5^; millions of people in the US have since received Paxlovid for acute COVID-19. Although studies have shown that Paxlovid is effective at reducing the risk of severe COVID-19, including hospitalization or death,^5^ limited research exists on whether it is effective in preventing Long COVID, and existing studies have found mixed results.^6–8^ Further, the association of Paxlovid with Long COVID risk is largely lacking in low-risk patients. A few low-risk patients were prescribed Paxlovid for acute illness,^1^ also providing the opportunity for examining the effectiveness of reducing Long COVID risk in those low-risk patients.

To date, the largest observational retrospective study on Paxlovid as a preventative treatment for Long COVID was conducted through an analysis of electronic health records (EHR) from 281,793 individuals with SARS-CoV-2 infection and at least 1 risk factor for progression to severe COVID-19 in the Veteran Affairs (VA) system. The study found that receipt of Paxlovid was associated with a reduced risk of Long COVID (hazard ratio 0.74; 95% CI, 0.72–0.77; absolute risk reduction, 4.51%; 95% CI, 4.01–4.99), as well as post-acute death or hospitalization.^9^ However, the study population, which was older and overwhelmingly white and male, was not representative of the general U.S. population, and the effectiveness of Paxlovid in preventing Long COVID remains unclear in demographically diverse populations.

In this study, we analyzed data from 29 EHR repositories of the national Patient-Centered Clinical Research Networks (PCORnet) as part of the NIH RECOVER initiative. We studied the association between Paxlovid treatment within 5 days of acute COVID-19 and the development of Long COVID, all-cause hospitalization, and all-cause mortality in the post-acute phase.

## Results

### Characteristics of the study population

**Figure 1:**
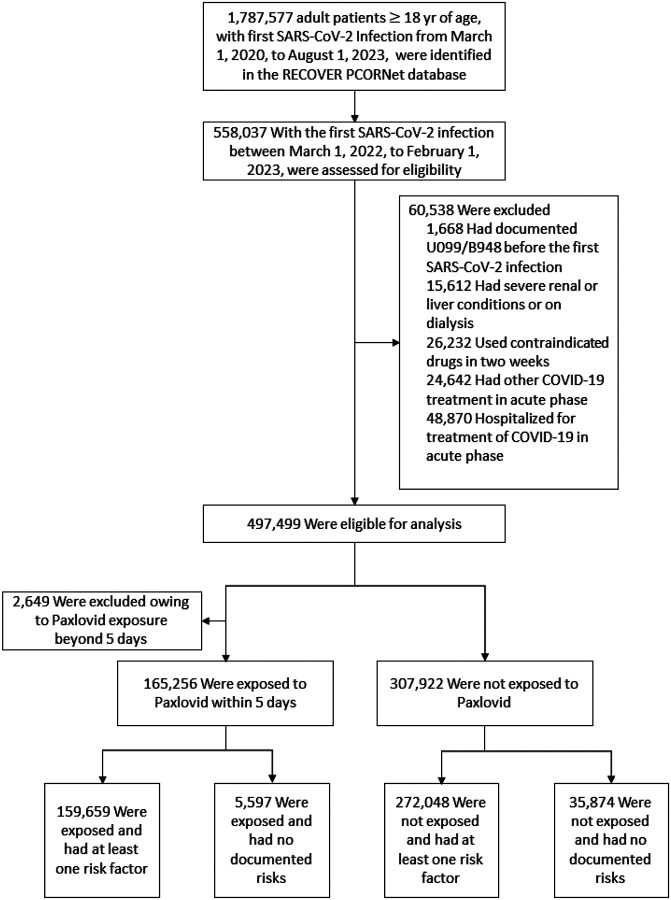
Selection of adult SARS-CoV-2 infected patients for evaluating the effectiveness of the acute prescription of Paxlovid on the PASC conditions, during the period from March 1, 2022, to February 1, 2023

Among 558,037 patients with their first SARS-CoV-2 infection between March 1, 2022, and February 1, 2023, 497,499 were eligible for the study (Fig. 1). Among eligible adult patients, 165,256 received Paxlovid within 5 days of infection and 307,922 did not. The baseline characteristics of the two groups are presented in Table 1.

The median age in the Paxlovid-treated group was older than the non-treated group (61 [interquartile range (IQR), 48–71 vs 50 [IQR 35–65]). Paxlovid-treated patients were more likely to be (self-reported) White than non-treated patients, and more likely to have a risk factor (e.g., cancer, hypertension, immune dysfunction, overweight, and obesity) for developing severe COVID-19 illness. They were less likely to be (self-reported) Black or Hispanic, or have substance use disorders.

Comparisons were conducted for the Paxlovid-treated group and non-treated group and all measured variables were well-balanced between corresponding comparisons as summarized in Figures S1 and S2 in the Supplementary Appendix. In addition, significant associations of exposure were not observed with negative outcomes, as summarized in Figures S3 and S4 in the Supplementary Appendix, suggesting little residual confounding.

### Paxlovid effectiveness

**Figure 2:**
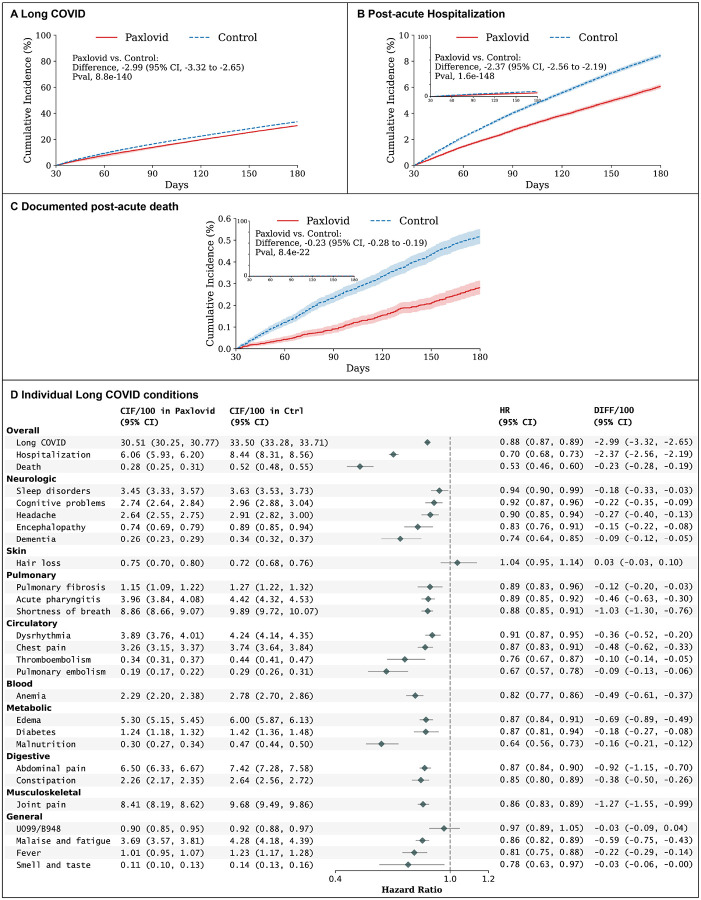
Absolute Risk, Risk Ratio, and Risk Difference of Long COVID in the Paxlovid group versus the Non-treated group among high-risk patients. A, the cumulative incidence of Long COVID; B, the cumulative incidence of post-acute death; C, the cumulative incidence of post-acute hospitalization; and D, the absolute risk, risk ratio, and risk difference of individual Long COVID at 180 days after the infection. Outcomes were ascertained 30 days after the first documented SARS-CoV-2 infection evidence until the end of the follow-up. The absolute risk, risk ratio, and risk difference were captured by the cumulative incidence (CIF), hazard ratio (HR), and the difference of cumulative incidence per 100 persons (DIFF/100), estimated at 180 days after the infection index date, respectively. Shaded areas represent pointwise 95% Confidence Intervals (95% CI).

At 180 days of follow-up, the estimated risk of Long COVID was 30.51 events per 100 persons (95% confidence interval (CI), 30.25 to 30.77) in the Paxlovid-treated group and 33.50 (95% CI, 33.28 to 33.71) in the non-treated group (Fig. 2). Compared to patients in the non-treatment group, Paxlovid-treated patients had a reduced risk of Long COVID, with a Hazard Ratio (HR) of 0.88 (95% CI, 0.87 to 0.89) and risk reduction of 2.99 events per 100 persons (95% CI, 2.65 to 3.32). Paxlovid treatment was also associated with a lower risk of all-cause hospitalization (HR, 0.70, 95% CI, 0.68–0.73; risk reduction of 2.37 events per 100 persons, 95% CI, 2.19 to 2.56), and all-cause death (HR, 0.53, 95% CI, 0.46 to 0.60; risk reduction of 0.23 events per 100 persons, 95% CI, 0.19 to 0.28) in the post-acute 180 days.

Paxlovid treatment was associated with a lower risk of incident Long COVID across systems as shown in Fig. 2D, including post-acute neurological conditions (sleep disorders, cognitive problems, headache, encephalopathy, dementia), post-acute pulmonary conditions (pulmonary fibrosis, acute pharyngitis, shortness of breath), post-acute blood conditions (anemia), post-acute metabolic conditions (edema, diabetes, malnutrition), post-acute digestive conditions (abdominal pain, constipation), post-acute musculoskeletal conditions (joint pain), and some general conditions in the post-acute phase (e.g., malaise and fatigue, fever, smell, and taste). There were no conclusive associations between Paxlovid and hair loss (HR, 1.04, 95% CI 0.95 to 1.14), or ICD-10 codes U0.99 or B94.8 representing unspecified Post COVID-19 conditions or sequelae of infectious illness (HR, 0.97, 95% CI 0.89 to 1.05).

**Figure 3:**
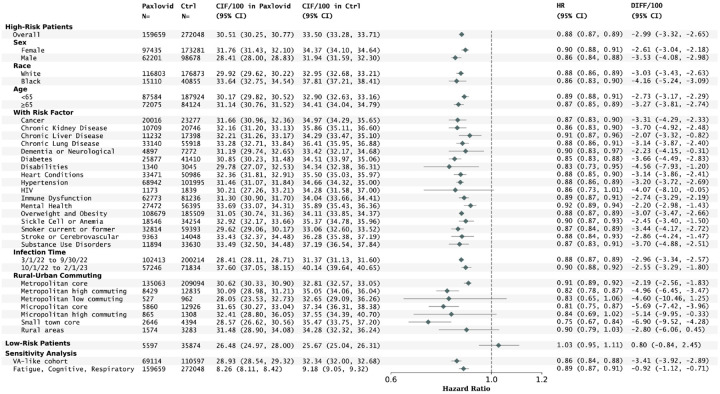
Risks of Long COVID in subgroups of high-risk patients (stratified by sex, race, age, coexisting conditions, infection time, and rural-urban areas), low risk patients, and sensitivity analyses by using different cohort and Long COVID definitions. The absolute risk, risk ratio, and risk difference were captured by the cumulative incidence (CIF), hazard ratio (HR), and the difference of cumulative incidence per 100 persons (DIFF/100), estimated at 180 days after the infection index date, respectively. Shaded areas represent pointwise 95% Confidence Intervals (95% CI).

Paxlovid treatment was also associated with a lower risk of incident Long COVID in different subpopulations stratified by sex, race, age, baseline risk conditions (except for pregnant females), infection time, and different rural-urban commuting areas as shown in Fig. 3. Relatively greater risk reductions were observed in males (3.53 events per 100 persons, 95% CI, 2.98 to 4.08) compared to females (2.61, 95% 2.18 to 3.04) and for individuals aged ≥ 65 (3.27, 95% CI, 2.74 to 3.81) compared to those under 65 (2.73, 95% CI, 2.29 to 3.17). Regarding the rural and urban areas, the greatest risk reduction was found in small towns (6.90 events per 100 persons, 95% CI, 4.28 to 9.52), followed by micropolitan (5.69, 95% CI, 3.96 to 7.42) and metropolitan patients (2.19, 95% CI, 1.83 to 2.56). The subgroup analyses for post-acute all-cause hospitalization or death are in Fig. S6 and S7 in the Supplementary Appendix. Results were broadly similar in sensitivity analyses that considered a secondary Long COVID definition with a focus on cognitive, fatigue, and respiratory conditions, and in a population with demographics similar to the Veteran Affairs study (87.8% male patients aged ≥60 years old or having any risk factors) (Fig. 3 and Fig. S8 in the Supplementary Appendix for the subgroup analyses).

However, in low-risk patients who didn’t have documented risk factors for severe COVID-19 illness or who were younger than 50, the association between Paxlovid treatment and incident Long COVID was inconclusive (HR, 1.03; 95% CI, 0.95–1.11).

## Discussion

In this clinical trial emulation of 497,499 individuals with diagnosed SARS-CoV-2 infection between March 1, 2022, and February 1, 2023, we found that Paxlovid treatment within 5 days of diagnosis was associated with a reduced risk of developing a range of post-acute conditions in various organ systems, among patients at high-risk for severe COVID-19 illness. Individuals treated with Paxlovid were also significantly less likely to be hospitalized or to die from any cause in the post-acute phase. These results suggest that Paxlovid may significantly reduce the risk of Long COVID and other downstream consequences of acute COVID-19 infection for high-risk non-hospitalized adults. Current guidelines recommend the use of Paxlovid for treating acute COVID-19;^5^ our findings suggest that its potential to reduce the risk of Long COVID conditions among high-risk patients should also be considered.

We did not, however, observe a reduction in Long COVID risk among patients who did not have risk factors for severe COVID-19. This finding is consistent with a recent randomized clinical trial that failed to show a significant benefit of Paxlovid in treatment of acute COVID-19 in low-risk populations.^10^ Taken together, this suggests limited utility of Paxlovid in low-risk patients, both for preventing short-term and long-term consequences of infection.^5^

This study has several strengths. First, it is the largest observational cohort study examining the impact of Paxlovid on Long COVID conditions.^9^ The study’s diverse demographic composition allows for more generalizable conclusions and allows for a more granular investigation of the effect of Paxlovid in various patient subgroups. Our study suggests that, compared to the veteran population,^9^ the beneficial effects of acute Paxlovid treatment on Long COVID can be extended to female patients, Black patients, and patients who live in metropolitan areas. Importantly there is no evidence of benefit of Paxlovid in preventing Long COVID for pregnant females and low-risk patients. Furthermore, though the beneficial effects span across different subgroups, greater risk reductions were observed in males compared to females, patients aged 65 years and older compared to those under 65, and rural patients compared to micropolitan and metropolitan patients. Second, this study included a broad set of Long COVID outcomes including post-acute incident conditions involving multiple organ systems, post-acute hospitalization, and death. Our results demonstrate that Paxlovid treatment is associated with reduced risk in most outcomes, especially in post-acute hospitalization, death, pulmonary embolism, dementia, and thromboembolism. One exception is the less severe outcome of hair loss which had no significant association with Paxlovid use. The benefits of Paxlovid treatment on Long COVID may be due to reduced acute severity of SARS-CoV-2 infection but differential effects might arise from distinct biologic mechanisms of different Long COVID subtypes.^1,11^

Our study also has limitations. First, this was an observational analysis, and patients were not randomized to treatment or control groups. However, the baseline covariates and negative outcome control analyses suggest relatively good balance between the groups; however, residual confounding may still exist. Second, we considered only incident conditions in the post-acute period, and did not examine worsening or prolonging of preexisting conditions after the COVID-19 infection. Third, the study used structured information in EHR, which may be incomplete and does not capture patients who do not seek or access care. Fourth, we estimated intention-to-treatment effects without modeling medication adherence during the acute phase; we could not determine adherence to Paxlovid treatment after prescription.

In conclusion, we conducted the largest observational study to date investigating the effectiveness of Paxlovid treatment within 5 days of SARS-CoV-2 infection in preventing Long COVID in a diverse patient population. Our study suggests that, among high-risk, nonhospitalized patients with COVID-19, treatment with Paxlovid during the acute illness was associated with reduced risk of Long COVID, post-acute hospitalization, and death. These benefits do not appear among low-risk patients.

## Methods

### Study Cohort

This observational analysis emulated a target trial^5,12^ to determine whether Paxlovid treatment for acute COVID-19 reduces the risk of Long COVID among nonhospitalized adults with COVID-19. We used data from 29 EHR repositories of the national Patient-Centered Clinical Research Network (PCORnet) as part of the NIH RECOVER initiative. Patients were identified as having acute COVID-19 if they had (a) positive SARS-CoV-2 polymerase-chain-reaction (PCR) or antigen laboratory tests; (b) the International Classification of Diseases, Tenth Revision, Clinical Modification (ICD-10-CM) diagnosis code U07.1 representing COVIID-19 diagnosis; or (c) Paxlovid (nirmatrelvir/ritonavir) or Remdesivir prescriptions. The index event was defined by the first documented evidence. The protocol is summarized in Table S1 in the Supplementary Appendix.

Key eligibility criteria included age 18 years or greater at the first confirmed SARS-CoV-2 infection between March 1st, 2022 and February 28th, 2023, interactions with the healthcare system in the past 3 years to 7 days before the index event, risk factors for progression to severe COVID-19 illness (≥ 50 years or having at least 1 underlying medical conditions associated with an increased risk of developing severe illness from COVID-19), no previous ICD-10-CM diagnosis codes of U0.99 (Post COVID-19 condition, unspecified) or B94.8 (Sequelae of other specified infectious and parasitic diseases), nonhospitalization for the treatment of COVID-19, and absence of severe liver disease, dialysis or severe renal impairment, use of other COVID-19 treatments including antiviral, antibody, or convalescent plasma treatments, or contraindication for Paxlovid. See the Supplementary Appendix Table S1 for the detailed specifications of protocols of the target trial and Table S2 for the study variables and associated time windows.

### Exposure

The exposure of interest was any Paxlovid prescription within 5 days of the SARS-CoV-2 infection. Comparison patients were those who didn’t receive Paxlovid treatment or other COVID-19 treatments in the acute infection. We classified patients based on their real-world treatment data and emulated randomization by adjusting for baseline confounders.

#### Outcomes

The outcomes of interest were incident post-acute sequelae of SARS-CoV-2 (PASC, or Long COVID), all-cause death, and all-cause hospitalization in the post-acute phase. The Long COVID definition is a rules-based computable phenotype leveraging ICD codes for 24 condition clusters.^11,13^ See the detailed condition clusters in the Supplementary Appendix-Long COVID definition section. All outcomes were ascertained from 30 days after the SARS-CoV-2 infection (time zero) to the day of the outcome of interest, death, 180 days after time zero, the day of the most recent interaction with the health system, or end of the study period (August 1, 2023), whichever occurred first. The death event, both in acute and post-acute phases, was treated as a competing event that precludes other outcomes of interest from occurring.

### Covariates

A broad range of potential confounders was considered, including sex, age, race and ethnicity, the Area Deprivation Index (ADI), body mass index (BMI), time of infection, smoking status, COVID-19 vaccine status, baseline clinical risk factors (cancer, chronic kidney disease, chronic liver disease, chronic lung diseases, cystic fibrosis, dementia or other neurological conditions, diabetes (type 1 or type 2), disabilities, heart conditions, HIV infection, immunocompromised condition or weakened immune system, mental health conditions, overweight and obesity, pregnancy, sickle cell disease or anemia, smoking, stroke or cerebrovascular disease, substance use disorders, tuberculosis)^14^ for developing severe COVID-19 illness, and additional covariates (coagulopathy, peripheral vascular disorders, seizure/epilepsy, weight loss, obstructive sleep apnea, Epstein-Barr and Infectious Mononucleosis, Herpes Zoster) might be associated with treatment and PASC outcomes. Covariates and assessment periods are summarized in Table S2 in the Supplementary Appendix

### Statistical Analysis

Baseline covariate balance was compared between the two exposure groups by standardized mean differences (SMD), with a difference of less than 0.1 considered to be balanced. The stabilized inverse probability of treatment weighting (IPTW) was used in the survival analysis to adjust for the baseline covariates.^15,16^ The propensity scores for different exposures were calculated with the regularized logistic regression with L2 norm, with exposure to Paxlovid or not as the dependent variable, and with all the baseline covariates as independent variables.^13,17^ Cumulative incidence curves for different groups were estimated with the Aalen-Johansen model in the reweighted population by considering death as a competing risk. The hazard ratios were estimated by the Cox survival model in the reweighted population, and two-sided 95% confidence intervals were calculated with the use of a robust variance estimator to account for stabilized IPTW weights. The absolute risk reduction was the difference in cumulative incidences at 180 days of follow-up between exposed and non-exposed groups. The nonparametric 1000-iteration bootstrapping estimation of risks, risk reductions, and percentile-based 95% confidence intervals were also calculated.

We conducted subgroup analyses according to sex (female or male), race (Black or White), age (< 65 or ≥65 years), different baseline risk factors, infection time, and areas with different rural-urban commuting patterns. In addition to investigating the at-risk patients, we also investigated patients without documented risk factors. Multiple sensitivity analyses were conducted to test the robustness of the results, including using a different Long COVID definition with a focus on fatigue, cognitive, and respiratory symptom clusters,^18^ cohorts similar to the US Veterans study (87.8% male patients aged ≥60 years old or having any risk factors).^9^ We further conducted two negative outcome control studies to explore the possibility of residual confounding.^19^

### Ethics

This study received Biomedical Research Alliance of New York (BRANY) institutional review board (IRB) approval #21-08-508. As part of the Biomedical Research Alliance of New York (BRANY IRB) process, the protocol has been reviewed in accordance with the institutional guidelines. The Biomedical Research Alliance of New York (BRANY) waived the need for consent and HIPAA authorization.

## Figures and Tables

**Table 1:** Population characteristics of adult SARS-CoV-2 infected patients for evaluating the effectiveness of the acute prescription of Paxlovid on the PASC conditions, during the period from March 1, 2022, to February 1, 2023

	Paxlovid treated SARS-CoV-2 Infected Patients. (N = 165,256)	No treated SARS-CoV-2 Infected Patients (N = 307,922)	SMD
**Characteristic**			
**Sex — no. (%)**			
Female	100,931 (61.1)	196,527 (63.8)	−0.06
Male	64,300 (38.9)	111,224 (36.1)	0.06
Other/Missing	25 (0.0)	171 (0.1)	−0.02
**Median age (IQR) — yr**	61 (48−71)	50 (35–65)	0.49
**Age group — no. (%)**			
18–24	3,649 (2.2)	26,292 (8.5)	−0.28
25–34	12,128 (7.3)	53,258 (17.3)	−0.31
35–49	30,936 (18.7)	74,274 (24.1)	−0.13
50–64	50,522 (30.6)	74,914 (24.3)	0.14
65+	68,021 (41.2)	79,184 (25.7)	0.33
**Race/Ethnicity — no. (%)**			
Asian Non-Hispanic	6,453 (3.9)	17,258 (5.6)	−0.08
Black or African American Non-Hispanic	15,125 (9.2)	43,307 (14.1)	−0.15
Hispanic or Latino Any Race	9,848 (6.0)	37,630 (12.2)	−0.22
White Non-Hispanic	116,020 (70.2)	175,712 (57.1)	0.28
Other Non-Hispanic	3,545 (2.1)	9,772 (3.2)	−0.06
Unknown	14,265 (8.6)	24,243 (7.9)	0.03
**No. of hospital visits in the past 3 year — no. (%)**			
Inpatient 0	138,148 (83.6)	261,619 (85.0)	−0.04
Inpatient 1–2	19,765 (12.0)	35,296 (11.5)	0.02
Inpatient > = 3	7,343 (4.4)	11,007 (3.6)	0.04
Outpatient 0	1,544 (0.9)	11,394 (3.7)	−0.18
Outpatient 1–2	7,899 (4.8)	26,481 (8.6)	−0.15
Outpatient > = 3	155,813 (94.3)	270,047 (87.7)	0.23
Emergency 0	130,061 (78.7)	223,574 (72.6)	0.14
Emergency 1–2	27,353 (16.6)	58,644 (19.0)	−0.07
Emergency > = 3	7,842 (4.7)	25,704 (8.3)	−0.15
**Median Area Deprivation Index (IQR) — rank**	30 (12−54)	37 (16–61)	−0.20
**BMI (IQR)**	29 (25−35)	29 (24–35)	0.03
**Documented Vaccination Status**			
Fully vaccinated	67,718 (41.0)	111,921 (36.3)	0.10
Partially vaccinated	26,333 (15.9)	41,717 (13.5)	0.07
No evidence	72,282 (43.7)	156,552 (50.8)	−0.14
**Patients in different Index time — no. (%)**			
03/22 – 06/22	48,541 (29.4)	117,644 (38.2)	−0.19
07/22 – 10/22	68,535 (41.5)	127,243 (41.3)	0.00
11/22 – 02/23	48,180 (29.2)	63,035 (20.5)	0.20
**Patient w/o documented risks—no. (%)**	5,597 (3.4)	35,874 (11.7)	−0.32
**Pregnant— no. (%)**	822 (0.5)	4,368 (1.4)	−0.09
**Patient with at least one risk factor— no. (%)**	158,837 (96.1)	267,680 (86.9)	0.33
**Individual Risk Factors — no. (%)**			
Cancer	20,016 (12.1)	23,277 (7.6)	0.15
Chronic kidney disease	10,709 (6.5)	20,746 (6.7)	−0.01
Chronic liver disease	11,232 (6.8)	17,398 (5.7)	0.05
Chronic lung disease	33,140 (20.1)	55,918 (18.2)	0.05
Cystic fibrosis	224 (0.1)	225 (0.1)	0.02
Dementia or other neurological conditions	4,897 (3.0)	7,272 (2.4)	0.04
Diabetes	25,877 (15.7)	41,410 (13.4)	0.06
Disabilities	1,340 (0.8)	3,045 (1.0)	−0.02
Heart conditions	33,471 (20.3)	50,986 (16.6)	0.10
Hypertension	68,942 (41.7)	101,995 (33.1)	0.18
HIV infection	1,173 (0.7)	1,839 (0.6)	0.01
Immune dysfunction	62,773 (38.0)	81,236 (26.4)	0.25
Mental health conditions	27,472 (16.6)	56,395 (18.3)	−0.04
Overweight and obesity	108,679 (65.8)	185,509 (60.2)	0.11
Sickle cell disease orthalassemia	18,546 (11.2)	34,254 (11.1)	0.00
Smoking current or former	32,814 (19.9)	59,393 (19.3)	0.01
Stroke or cerebrovasculardisease	9,363 (5.7)	14,048 (4.6)	0.05
Substance use disorders	11,894 (7.2)	33,630 (10.9)	−0.13
Tuberculosis	77 (0.0)	199 (0.1)	−0.01

a. The SARS-CoV-2 positive patients were identified by a) positive SARS-CoV-2 polymerase-chain-reaction (PCR) or antigen laboratory tests; b) the ICD-10-CMdiagnosis code U07.1 representing COVIID-19 diagnosis; or c) Paxlovid (nirmatrelvir/ritonavir) or Remdesivir prescriptions. The first documented evidence was defined as the index event. IQR denotes the interquartile range. The percentage may not sum up to 100 because of rounding. ADI, the Area Deprivation Index. BMI, Body Mass Index. b. A standardized mean difference (SMD) of > 0.10 or <−0.10 indicates an important effect size difference between the two samples, otherwise, no significant difference is assumed. c. Pregnant patients were included when SARS-CoV-2 infection occurred during pregnancy.

## Data Availability

The de-identified data used in this analysis are considered the domain of the contributing health systems. These data were shared with the RECOVER research program under data-sharing agreements. Requests for deidentified data can be considered through the RECOVER research program but would need the approval of the contributing health systems.
